# *Scandinavium goeteborgense* gen. nov., sp. nov., a New Member of the Family *Enterobacteriaceae* Isolated From a Wound Infection, Carries a Novel Quinolone Resistance Gene Variant

**DOI:** 10.3389/fmicb.2019.02511

**Published:** 2019-11-05

**Authors:** Nachiket P. Marathe, Francisco Salvà-Serra, Roger Karlsson, D. G. Joakim Larsson, Edward R. B. Moore, Liselott Svensson-Stadler, Hedvig E. Jakobsson

**Affiliations:** ^1^Institute of Marine Research, Bergen, Norway; ^2^Department of Infectious Diseases, Sahlgrenska Academy, University of Gothenburg, Gothenburg, Sweden; ^3^Centre for Antibiotic Resistance Research, University of Gothenburg, Gothenburg, Sweden; ^4^Department of Clinical Microbiology, Culture Collection University of Gothenburg, Sahlgrenska University Hospital and Sahlgrenska Academy, University of Gothenburg, Gothenburg, Sweden; ^5^Microbiology, Department of Biology, University of the Balearic Islands, Palma de Mallorca, Spain; ^6^Nanoxis Consulting AB, Gothenburg, Sweden

**Keywords:** novel genus, novel species, *Enterobacteriaceae*, quinolone resistance, wound infection, antibiotic resistance, phylogenomics, taxonomy

## Abstract

The family *Enterobacteriaceae* is a taxonomically diverse and widely distributed family containing many human commensal and pathogenic species that are known to carry transferable antibiotic resistance determinants. Characterization of novel taxa within this family is of great importance in order to understand the associated health risk and provide better treatment options. The aim of the present study was to characterize a Gram-negative bacterial strain (CCUG 66741) belonging to the family *Enterobacteriaceae*, isolated from a wound infection of an adult patient, in Sweden. Initial phenotypic and genotypic analyses identified the strain as a member of the family *Enterobacteriaceae* but could not assign it to any previously described species. The complete 16S rRNA gene sequence showed highest similarity (98.8%) to four species. Whole genome sequencing followed by *in silico* DNA-DNA similarity analysis and average nucleotide identity (ANI) analysis confirmed that strain CCUG 66741 represents a novel taxon. Sequence comparisons of six house-keeping genes (16S rRNA, *atpD*, *dnaJ, gyrB*, *infB*, *rpoB*) with those of the type strains of the type species of related genera within the family *Enterobacteriaceae* indicated that the strain embodies a novel species within the family. Phylogenomic analyses (ANI-based and core genome-based phylogeny) showed that strain CCUG 66741 forms a distinct clade, representing a novel species of a distinct, new genus within the family *Enterobacteriaceae*, for which the name *Scandinavium goeteborgense* gen. nov., sp. nov. is proposed, with CCUG 66741^T^ as the type strain (= CECT 9823^T^ = NCTC 14286^T^). *S. goeteborgense* CCUG 66741^T^ carries a novel variant of a chromosomally-encoded quinolone resistance gene (proposed *qnrB96*). When expressed in *Escherichia coli*, the *qnrB96* gene conferred five-fold increase in minimum inhibitory concentration against ciprofloxacin. This study highlights the importance and the utility of whole genome sequencing for pathogen identification in clinical settings.

## Introduction

The family *Enterobacteriaceae* is a large and taxonomically diverse group of Gram-negative rod-shaped bacteria within the class *Gammaproteobacteria* and order *Enterobacteriales* that includes many common human pathogens (Hata et al., 2016). Numerous genera within the *Enterobacteriaceae* family are recognized to be polyphyletic, based on the 16S rRNA gene (Brady et al., 2008; Kämpfer et al., 2015; Alnajar and Gupta, 2017), hence multilocus sequence analyses (MLSA) based on several house-keeping gene sequences have been proposed for reliable classification of isolates and strains within this family (Brady et al., 2013). The advent of next-generation sequencing and whole genome sequencing (WGS) analysis has significantly impacted the taxonomic classification of *Enterobacteriaceae* members. Recently, using whole genome-based phylogeny and taxonomy, some members of this family have been reclassified into separate and novel families (e.g., *Budviciaceae*, *Erwiniaceae*, *Hafniaceae*, *Morganellaceae*, *Pectobacteriaceae*, *Yersiniaceae*), belonging to the order *Enterobacterales* (Adeolu et al., 2016). Nevertheless, many species within *Enterobacteriaceae* are important human pathogens usually carrying transferable antibiotic resistance markers (Carattoli, 2009). Indeed, the World Health Organization has included carbapenem-resistant and extended-spectrum beta-lactamase-producing *Enterobacteriaceae* within the Priority 1 (critical) group, in the list of priority pathogens for research and development of new antibiotics (World Health Organization [WHO], 2017). Thus, taxonomic description of novel members of the family is important in order to understand associated health risks and to prevent spread of infections.

The aim of the current study was to characterize a Gram-negative bacterial strain (CCUG 66741) isolated from a wound infection of an adult patient in Sweden. We here present a novel genus in the family *Enterobacteriaceae*: *Scandinavium goeteborgense* gen. nov., sp. nov., with reference to taxonomy, genome analysis and identification of a novel variant of quinolone resistance gene (*qnrB96*).

## Results

### Strain Isolation and Characterization

Strain CCUG 66741^T^ was isolated from a wound infection of an adult patient, which was sampled in Kungälv, Sweden. The colonies on Columbia Blood Agar, after 24 h of incubation at 30°C, were 2–3 mm in size, circular, moist, and smooth. Cells were Gram-negative, rod-shaped, around 2 μm in length and around 1 μm in width. The strain was typed using MALDI-TOF MS at the Department of Clinical Microbiology of the Sahlgrenska University Hospital (Gothenburg, Sweden), using the IVD-Database Knowledge Base v2 (bioMérieux, Marcy-l’Étoile, France). The typing analysis could not assign strain CCUG 66741^T^ to any species, with *Escherichia vulneris* being identified as the most similar species, although with a confidence value of 74.4%, which is below the 90% threshold for species identification recommended by the manufacturer. API^®^ ID 32E metabolic profiling with StrainMatcher, indicated that the most closely-related species (i.e., those with lowest StrainMatcher scores) were *Buttiauxella warmboldiae*, *Buttiauxella noackiae*, *Buttiauxella izardii*, and *Enterobacter cancerogenus* (Table 1). These results did not allow reliable assignation of the strain CCUG 66741^T^ to any species.

**TABLE 1:** Results of the API^®^ ID 32E analysis (bioMérieux).

**Strain**	**StrainMatcher score**
*Buttiauxella warmboldiae* CCUG 35512^T^	20
*Buttiauxella noackiae* CCUG 35511^T^	22
*Buttiauxella izardii* CCUG 35510^T^	28
*Enterobacter cancerogenus* CCUG 25231^T^	38

Partial sequencing of 16S rRNA gene and *dnaJ* was performed, which yielded sequences of 621 and 711 bp, respectively. Comparative sequence analysis of the 16S rRNA partial gene sequence with EMBL-EBI Nucleotide Database and the FASTA search tool (Madeira et al., 2019), showed a highest similarity value of 98.7% with *Buttiauxella ferragutiae* DSM 9390^T^, *Buttiauxella agrestis* DSM 4586^T^, *Buttiauxella izardii* DSM 9397^T^, and *Buttiauxella noackiae* DSM 9401^T^ (GenBank accession numbers: AJ233402, AJ233400, AJ233404, and AJ233405, respectively). The analysis of the *dnaJ* partial sequence showed the highest similarity value of 86.3% with *Leclercia adecarboxylata* GTC 1267^T^ (GenBank accession number: AB272662). Analyses performed by Pham et al. (2007) showed that intraspecific variation of *dnaJ* within the family *Enterobacteriaceae* ranges from 0 to 8.3%. Therefore, 14% sequence divergence compared with *L. adecarboxylata* GTC 1267^T^ was considered not enough to assign strain CCUG 66741^T^ to this species. The similarity values of the 16S rRNA and *dnaJ* partial gene sequences, thus, suggested that strain CCUG 66741^T^ could represent a novel taxon within the family *Enterobacteriaceae*.

The antibiotic sensitivity pattern revealed that the strain is sensitive to several antibiotics including cefotaxime, co-trimaxozole, meropenem, tetracycline, tobramycin, and ciprofloxacin but resistant to ampicillin. Additional metabolic and other phenotypic characteristics of strain CCUG 66741^T^ were determined, including a variety of biochemical tests presented in Supplementary Table S1. The strain CCUG 66741^T^ utilizes several sugars including glucose, galactose, xylose, mannose and inulin while it does not utilize lactose, raffinose, sorbose, starch, glycerol, and glycogen. The strain CCUG 66741^T^ is MR positive, produces acetoin and does not utilize citrate (Supplementary Table S1).

Cell fatty acid-fatty acid methyl ester (CFA-FAME) analysis of strain CCUG 66741^T^ determined the major CFAs to be the unsaturated fatty acid, C_16__:__1_ω7*c* (35.0%), saturated fatty acid, C_16__:__0_ (19.0%), and C_14__:__0_ 3OH/16:1 ISO I (18.9%), while other CFAs observed included C_12__:__0_ (5.6%), C_14__:__0_ (7.7%), C_15__:__0_ (2.3%), C17:0 CYCLO (4.6%), and C_18__:__1_ω7*c*/12t/9t (7.0%).

### Whole Genome Sequencing and Genome Related Indices

The genomes of strain CCUG 66741^T^ and two other type strains of the family *Enterobacteriaceae* that did not have publicly available genome sequences in DDBJ/ENA/GenBank (*Buttiauxella izardii* CCUG 35510^T^ and *Lelliottia nimipressuralis* CCUG 25894^T^) were sequenced in this study. Genome assembly statistics are presented in Supplementary Table S2. The genome sequence of strain CCUG 66741^T^ comprises 192 contigs with a total length of 4,539,908 bp, G + C content of 54.3% and 4,384 coding sequences.

A complete 16S rRNA gene sequence (1,552 bp) was recovered from the genome sequence of strain CCUG 66741^T^. The sequence analysis revealed ≥ 98.7% similarity, as determined by EzBiocloud (Yoon et al., 2017), to the type strains of four species of different genera within the family *Enterobacteriaceae* and the type strain of *Pantoea rodasii*, a member of family *Erwiniaceae* (Supplementary Table S3). *In silico* DNA-DNA similarity values determined between the genome sequence of strain CCUG 66741^T^ and the genome sequences of the type strains of the five species with 16S rRNA gene sequence similarity values ≥ 98.7% were lower than 25%, far below the 70% species delineation threshold (Goris et al., 2007). Additionally, the average nucleotide identities based on BLAST (ANIb) values between the genome sequences of the five type strains and the genome sequence of strain CCUG 66741^T^ were lower than 80%, well below the threshold accepted (95%) for defining species (Richter and Rosselló-Móra, 2009; Supplementary Table S3).

### Additional Related Strain Genome Sequences

BLAST analyses, using house-keeping gene sequences of strain CCUG 66741^T^, revealed two closely-related strains: BIGb0156 (GenBank accession number: SNVX00000000) and YR018 (GenBank accession number: QKME00000000). The 16S rRNA gene sequences similarity values between strain CCUG 66741^T^ and strains BIGb0156 and YR018 were 99.9%, while ANIb values were 97.70% and 96.04%, respectively, thus confirming that all three genome sequences belong to the same species.

Strain BIGb0156 was isolated from rotting apple in Orsay, France, in 2009 (Samuel et al., 2016); the genome sequence has a total length of 4,755,497 bp, G + C content of 54.4% and 4,513 coding sequences. Strain YR018 was isolated from *Populus* rhizosphere in Tennessee, United States; the genome sequence has a total length of 4,498,002 bp, G + C content of 54.8% and 4,181 coding sequences.

### Multilocus Sequence Analysis (MLSA)

**FIGURE 1:**
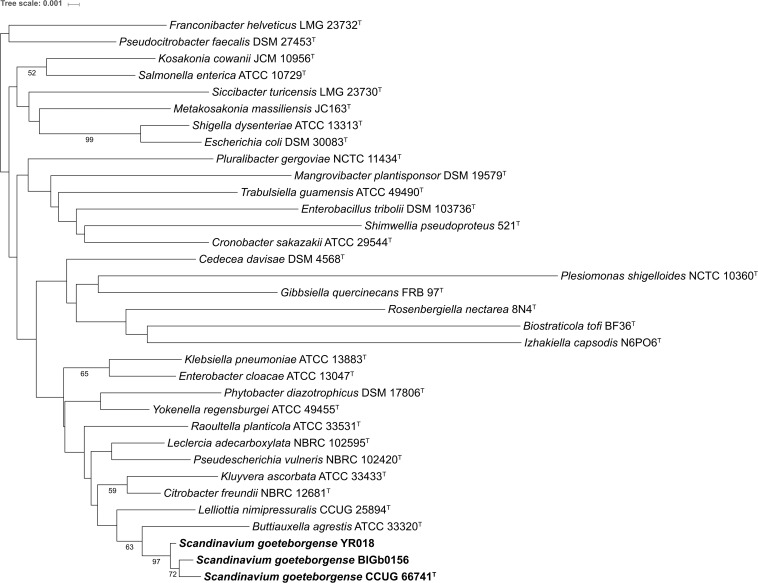
Phylogenetic tree based on the 16S rRNA gene sequences of the type strains of the type species of the genera with validly published names within the family *Enterobacteriaceae* and strains of *S. goeteborgense*. The distances were calculated with the Jukes-Cantor method and the tree was constructed using the neighbor-joining method. The numbers at the nodes indicate the bootstrap values (in percentage) from 1,000 replicates. Only bootstrap percentages ≥ 50% are shown.

The family *Enterobacteriaceae* currently comprises 32 genera with validly published names. The genome sequence of the type strain of the type species of 30 of these genera were obtained from GenBank^1^. Complete or nearly-complete (>1,400 bp) 16S rRNA gene sequences were recovered for 31 of the 32 type strains of type species; no sequences were available in GenBank for the type strain of *Saccharobacter fermentatus*. Furthermore, the type strain is not available at any public strain collection (Yarza et al., 2013). Hence *S. fermentatus* was not included in our analysis. The GenBank accession numbers of the obtained genome sequences and 16S rRNA gene sequences are presented in Supplementary Table S4. According to 16S rRNA gene sequence-based phylogeny, *Buttiauxella* and *Lelliottia* were the most closely-related genera to strain CCUG 66741^T^ (Figure 1).

In parallel, complete sequences of other house-keeping genes (*atpD*, *dnaJ*, *infB*, *gyrB*, *rpoB*) were extracted from the genome sequences of the type strains of 30 type species and from strain CCUG 66741^T^. These house-keeping genes showed sequence similarity values ranging from 73.8 to 93.7%, suggesting that the strain represents a novel genus within *Enterobacteriaceae* (Supplementary Table S5). The MLSA-based phylogenetic tree showed that strain CCUG 66741^T^ forms a separate distinct clade compared to other genera, while strains BIGb0156 and YR018 fall in the same clade as the strain CCUG 66741^T^ (Supplementary Figure S1), thus, suggesting that strain CCUG 66741^T^ may represent a novel genus within the family *Enterobacteriaceae.*

### Whole-Genome Sequence ANIb Dendrogram

**FIGURE 2:**
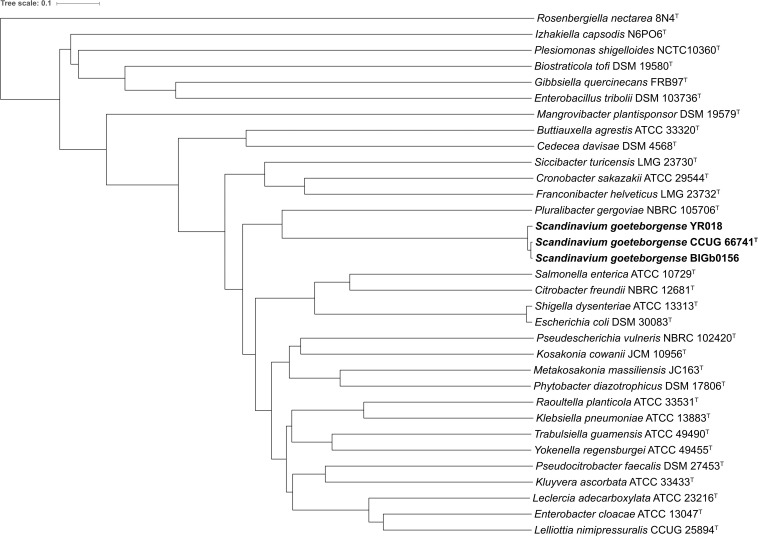
Dendrogram based on the ANIb values calculated between all available genome sequences (“all-vs.-all”) of type strains of the type species of the genera within the family *Enterobacteriaceae* and the genome sequences of strains of *S. goeteborgense*.

The ANIb values between all (“all-vs.-all”) 31 genome sequences (strain CCUG 66741^T^ and the type strains of the 30 type species) were calculated and used to construct a dendrogram. The ANIb values ranged from 67.9% (between *Pluralibacter gergoviae* NBRC 105706^T^ and *Rosenbergiella nectarea* 8N4^T^) to 96.4% (between *Escherichia coli* DSM 30083^T^ and *Shigella dysenteriae* ATCC 13313^T^). The ANIb values between the genome sequence of strain CCUG 66741^T^ and all other type strains ranged from 68.4% (*R. nectarea* 8N4^T^) to 79.2% (*P. gergoviae* NBRC 105706^T^), suggesting that *P. gergoviae* is the most closely related genus to strain CCUG 66741^T^. The dendrogram constructed using the matrix of ANIb values, confirmed the same observation (Figure 2).

### Core Genome-Based Phylogenomic Analysis

**FIGURE 3:**
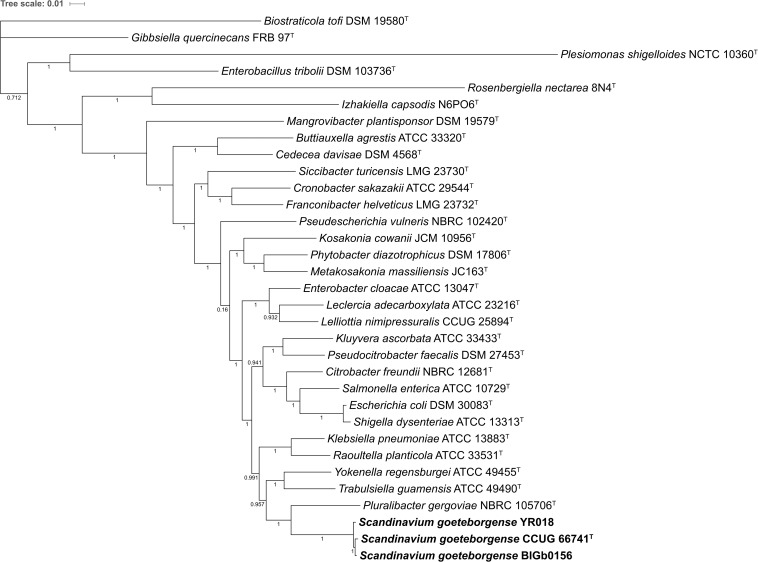
Phylogenomic tree based on the core genome analysis of all available genome sequences of the type strains of type species of genera with validly published names of the family *Enterobacteriaceae* and genome sequences of strains of *S. goeteborgense*. The numbers at the nodes indicate the SH-aLRT support values.

A core genome-based phylogenomic analysis was carried out with the 30 available genome sequences of type strains of type species of genera with validly published names of the family *Enterobacteriaceae*, in order to determine the relative taxonomic identity of strain CCUG 66741^T^. The core genome was calculated with a clustering criterion of 70% identity over 70% of the sequence length. A total of 131,456 amino acids encoded by 391 single-copy shared genes were used to construct a core genome-based phylogenetic tree. Strain CCUG 66741^T^, together with strains BIGb0156 and YR018, formed a distinct and separate clade from the other genera, thus indicating that they represent a novel genus within the family. *Pluralibacter* was the most closely related genus to strain CCUG 66741^T^ (Figure 3).

### Virulence Genes and Characterization of a Novel *qnrB* Variant

The genome of strain CCUG 66741^T^ carries several genes encoding putative virulence factors, including a type VI secretion system and associated effector proteins, hemolysins, virulence factor *VirK*, cytotoxin, enterotoxin and a protein similar to the Shiga toxin subunit A, thus indicating pathogenicity potential. The strain was predicted to be a human pathogen (probability 0.83) by PathogenFinder (Cosentino et al., 2013).

**FIGURE 4:**
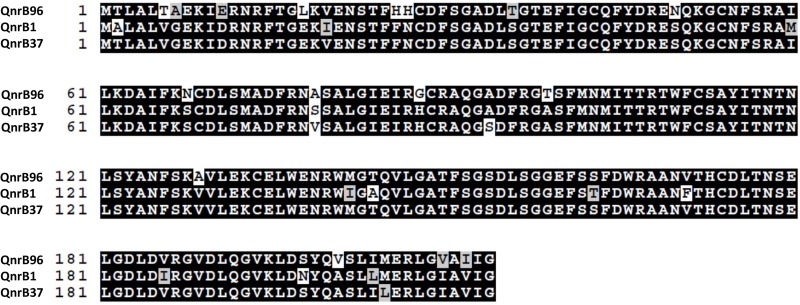
Sequence alignment of the novel Qnr variant (QnrB96) with QnrB1 and QnrB37, respectively. Black color indicates consensus. Multiple sequence alignment obtained using BoxShade (https://embnet.vital-it.ch/software/BOX_form.html).

Interestingly, a novel variant of *qnrB* gene (proposed *qnrB96*) was detected in the genome. The phylogenetic tree constructed, using all known *qnrB* variants shows *qnrB96* to be a new allele (Supplementary Figure S2). The QnrB96 protein was phylogenetically close to QnrB37 (92% sequence similarity), with 18 aa substitutions (Figure 4). The gene is located on a contig of 102 kb (locus tag: A8O29_06680) flanked by DNA gyrase B and a hypothetical protein without the presence of any transposable element in the contig, suggesting chromosomal localization of the gene. Consistent with other *qnrB* alleles, a LexA-binding sequence of sequence of CTGTATAAAAAAACAG was present 11 nucleotides upstream of the start codon of *qnrB96* (Ribeiro et al., 2015). The *qnrB96* gene and *qnrB1* conferred reduced sensitivity against ciprofloxacin (five and eight-fold increase in minimum inhibitory concentration, respectively) when expressed in *Escherichia coli*, thus confirming functionality.

## Discussion

Using a polyphasic approach by combining phenotypic and phylogenomic analyses, we describe *Scandinavium goeteborgense*, a novel species of a novel genus within the family *Enterobacteriaceae*. In recent years, WGS has been proposed as a high-resolution tool for taxonomic studies of prokaryotes (Chun and Rainey, 2014; Ramasamy et al., 2014; Mahato et al., 2017; Chun et al., 2018). Our study demonstrates discrepancies with single-gene-based estimates of phylogenetic relationships, such as 16S rRNA gene sequence-based analyses, and the importance of WGS for determining taxonomic relationships and pathogen characterization in the clinics. We also describe a novel functional variant of *qnrB* (*qnrB96*). When expressed in *E. coli*, it conferred five-fold increase in minimum inhibitory concentration against ciprofloxacin. The *qnr* genes encode pentapeptide repeat proteins that protect DNA gyrase and topoisomerase IV from the action of quinolones, but usually do not confer complete clinical resistance (Strahilevitz et al., 2009). The *qnr* genes were grouped into six families (*qnrA*, *qnrB*, *qnrC*, *qnrD*, *qnrS*, *qnrVC*) and recently *qnrE* has been proposed as a new *qnr* family (Strahilevitz et al., 2009; Albornoz et al., 2017). Among these, *qnrB* is the most diverse, with the largest number of variants, and mainly detected in *Enterobacteriaceae* strains (Ribeiro et al., 2015). Phylogenetic clustering of *qnrB* alleles forms several distinct lineages, suggesting multiple origins of *qnrB* alleles (Supplementary Figure S2). Studies have proposed *Citrobacter* spp. as the origin of *qnrB* genes, mainly based on the chromosomal location and the apparent absence of mobile genetic elements in the immediate genetic environment of *qnrB* genes (Jacoby et al., 2011; Ribeiro et al., 2015). Phylogeny of QnrB suggests that the *qnrB96* gene may be derived from *Citrobacter* or evolved independently from a common progenitor.

The taxonomy of family *Enterobacteriaceae* has not been straight-forward. Several genera and species belonging to this family have been reclassified through the years (Stephan et al., 2014; Hata et al., 2016; Li et al., 2016). Recently, extensive revisions of the species belonging to the genus *Enterobacter* have been made, resulting in proposals of new genera within the family (Brady et al., 2013). Widely used gene markers, such as 16S rRNA genes, are often ineffective for genus-level phylogenetic analyses within *Enterobacteriaceae* (Kämpfer et al., 2005; Young and Park, 2007; Brady et al., 2008; Naum et al., 2008). The 16S rRNA gene sequence of strain CCUG 66741^T^ showed high (98.8%) similarity to species of different genera within the family, as well as to the genus *Pantoea* (member of the family *Erwiniaceae*), not allowing the assignment of strain CCUG 66741^T^ to any known species within a genus of *Enterobacteriaceae.* To overcome the low resolution power of the 16S rRNA gene sequences, MLSA, using four different house-keeping gene sequences (*atpD, gyrB, infB*, and *rpoB)* has been applied, leading to descriptions of novel genera within the family (Brady et al., 2013). Additionally, the house-keeping gene *dnaJ* has also been proposed for performing phylogenetic studies within the family *Enterobacteriaceae*. The strain CCUG 66741^T^ showed highest sequence similarities to different species for the different house-keeping genes analyzed. The similarity values ranged from 80.7 to 93.7% for *atpD*, 74.2 to 86.3% for *dnaJ*, 73.1 to 87.6% for *infB*, 74.8 to 87.2% for *gyrB*, and 82.3 to 91.1% for *rpoB* (Supplementary Table S5). Low sequence identities of these house-keeping genes to the respective genes of the type species of genera with validly published names suggest that the strain CCUG 66741^T^ belongs to a novel taxon within the family.

WGS has been proposed as a tool for pathogen detection and characterization in the clinics (Quainoo et al., 2017). The usefulness of WGS and phylogenomic methods in taxonomic resolution of the family *Enterobacteriaceae* has been previously demonstrated (Adeolu et al., 2016; Alnajar and Gupta, 2017). Recently, a novel genus was proposed based on phenotypic and phylogenomic analysis (not validated yet) (Potter et al., 2018). We determined the genome sequence of strain CCUG 66741^T^ in order to resolve the taxonomic identity of the strain. According to recommendations by Chun et al. (2018), five species which showed more than 98.7% identity to 16S rRNA gene sequence of the strain CCUG 66741^T^ were selected for calculation of overall genome related indices like *in silico* DNA-DNA similarity and ANIb (Chun et al., 2018). However, the genome sequence of one of them (i.e., the type strain of *Buttiauxella izardii*) was not available in any public database. To be able to perform genomic comparisons, we carried out whole genome sequencing of *B. izardii* CCUG 35510^T^ (GenBank accession number: QZWH00000000). This genome sequence, increased the genomic coverage of the type strains of the family *Enterobacteriaceae* in public databases, which are the basis of numerous sequence-based taxonomic studies. Overall genome related indices between strain CCUG 66741^T^ and the five closely related species based on 16S rRNA gene sequence were substantially lower than the species delineation thresholds (*in silico* DNA-DNA hybridization values were < 25%, species delineation threshold ≥ 70%; ANIb values were < 80%; species assignment threshold ≥ 95%), confirming that strain CCUG 66741^T^ represents a novel species and, possibly, a novel genus (Goris et al., 2007; Chun et al., 2018).

We compared the genome sequence of the strain CCUG 66741^T^ with the genome sequences of the type strains of type species of genera with validly published names that currently form the family *Enterobacteriaceae*. According to the “List of Prokaryotic Names with Standing in the Nomenclature”^2^ and the “Prokaryotic Nomenclature Up-to-date” of the *Deutsche Sammlung von Mikroorganismen und Zellkulturen*^3^, the family *Enterobacteriaceae* encompasses, at least, 67 recognized genera (October 2018). However, 33 of them were recently reclassified into novel families (e.g., *Yersinia* in the family *Yersiniaceae*) and two were reclassified into existing genera (e.g., *Calymmatobacterium granulomatis*, type species and only species of the genus *Calymmatobacterium*, was reclassified as *Klebsiella granulomatis*) (Adeolu et al., 2016). Thus, only 32 genera of the family *Enterobacteriaceae* were considered in this study. Of these 32 genera, 29 had genome sequences for the type strains of the type species publicly available, one was sequenced in this study, whereas two lacked genome sequences. Consistent with the observations of MLSA-based phylogeny and ANIb dendogram, core genome phylogeny showed that the strain CCUG 66741^T^ formed a distinct clade, separate from all other genera with validly published names. The WGS-based approaches are robust, reliable and have a much higher resolving power compared to conventional, single-gene genotypic methods. Additionally, they bypass the risk of reaching erroneous conclusions due to horizontally transferred genes (Quainoo et al., 2017; Schürch et al., 2018). The ANIb, as well as overall genome related indexes, can be used to determine if two strains belong to the same species, but they do not provide enough resolution for working with taxa at a higher rank than species (Chun et al., 2018). Thus, core genome based phylogeny is recommended for defining taxa above the species level (Chun et al., 2018). MLSA-based phylogeny and ANIb dendrogram, as well as the core genome-based phylogenomic clustering, show that strain CCUG 66741^T^, together with strains BIGb0156 and YR018, forms a distinct clade. Thus, we conclude that strain CCUG 66741^T^ belongs to a novel genus within the family *Enterobacteriaceae*.

One limitation of our study is that we have analyzed only one strain. We tried to counteract this limitation by finding two additional strains belonging to the proposed novel taxon, by screening publicly available genome sequences. Even though this is not optimal from a taxonomic perspective, novel genera in family *Enterobacteriaceae* including *Chania* and *Nissabacter* (proposed reclassification to family *Yersiniaceae)* have previously been established from single strain investigations (Ee et al., 2016; Mlaga et al., 2017). In any case, our results confirm that strain CCUG 66741^T^ represents a novel taxonomic position within the family *Enterobacteriaceae*, representing a novel genus and species, for which the name *Scandinavium goeteborgense* gen. nov., sp. nov. is proposed, thus extending the diversity of clinically-relevant species belonging to this family.

### Description of *Scandinavium* gen. nov.

*Scandinavium* (Scan.di.na’vi.um. N.L. neut. n. *Scandinavium* genus named after Scandinavia: the European peninsula where the type strain of the type species was isolated and characterized).

Cells are Gram-negative, rod shaped, approximately 2 μm in length and 1 μm in width, motile and facultative anaerobic. The colonies on Columbia Blood Agar are 2–3 mm in size, circular, moist and smooth. Optimum temperature for growth is between 30–37°C, with no growth observed at 4°C and 42°C. Negative for gelatinase, urease and oxidase. Does not utilize citrate. Ferments glucose (acid and no gas), but not lactose. Utilizes galactose, xylose, maltose, mannitol and arabinose while does not utilize sucrose, melibiose, raffinose and starch. The major fatty acids are C_16__:__1_ω7*c*, C_16__:__0_, and C_14__:__0_ 3OH/C_16__:__1__ISO_, while C_14__:__0_, C_18__:__1_ω7*c*/12t/9t, and C_12__:__0_ are present in lower amount.

Members belong to class *Gammaproteobacteria*, order *Enterobacterales* and family *Enterobacteriaceae*. The type species of the genus is *Scandinavium goeteborgense*.

### Description of *Scandinavium goeteborgense* sp. nov.

*Scandinavium goeteborgense* (goe.te.borg.en’se. N.L. neut. adj. *goeteborgense* pertaining to Göteborg, the city on the Swedish west coast, where it was identified as a novel species).

All the features of genera apply for the species. The 16S rRNA gene sequence showed highest identity to *Enterobacter ludwigii* EN-119^T^, *Enterobacter roggenkampii* EN-117^T^, *Leclercia adecarboxylata* NBRC 102595^T^, and *Pantoea rodasii* LMG 26273^T^ (98.8%). G + C percentage of strain CCUG 66741^T^ is 54.3%. The type strain is CCUG 66741^T^ (= CECT 9823^T^ = NCTC 14286^T^).

## Conclusion

The present study describes a novel genus and species belonging to the family *Enterobacteriaceae, Scandinavium goeteborgense* gen. nov., sp. nov., isolated from a human wound infection, illustrating the usefulness and high-resolution of core genome-based analyses for the description of bacterial taxa above the species level. Characterization of a novel functional quinolone resistance gene variant (*qnrB96*) encoded in the genome of *S. goeteborgense* CCUG 66741^T^ increases the current understanding of pathogenic genera belonging to the family *Enterobacteriaceae* and associated resistance determinants. It also highlights the importance of using WGS as a tool for characterization of pathogens and detection of novel antibiotic resistance determinants especially in clinics.

## Materials And Methods

### Strain Isolation

Strain CCUG 66741^T^ (= CECT 9823^T^ = NCTC 14286^T^) was isolated from a wound infection of an adult patient. The wound was sampled in Kungälv, Sweden, in December 2014, and the isolate was obtained at the Department of Clinical Microbiology of the Sahlgrenska University Hospital, in Gothenburg, Sweden, after cultivation on Columbia Blood Agar Base plus 5% defibrinated horse blood (prepared by the Substrate Unit, Department of Clinical Microbiology, Sahlgrenska University Hospital) grown at 37°C, aerobically.

### MALDI-TOF MS

The bacterial strain was typed using Matrix-Assisted Laser Desorption/Ionization-Time Of Flight Mass Spectrometry (MALDI-TOF MS). For MALDI-TOF MS analysis, strain CCUG 66741^T^ was cultivated overnight on Columbia Blood Agar at 30°C, aerobically. Fresh biomass was spotted in two replicates on a target plate (bioMérieux) and covered with 1 μl VITEK MS-CHCA matrix (α-Cyano-4-hydroxycinnamic acid, bioMérieux). The sample was analyzed, using a VITEK MS instrument (bioMérieux), following the manufacturer’s recommendations. The IVD-Database Knowledge Base v2 was used (bioMérieux). Positive identification was considered if the confidence value was > 90%, according to manufacturer’s instructions.

### Growth Assays and Biochemical Tests

Further characterization of strain CCUG 66741^T^ was performed at the typing laboratory of the Culture Collection University of Gothenburg (CCUG). Colony morphology was observed after growth on Columbia Blood Agar at 30°C, aerobically, for 24 h. Cell morphology and size was determined by Gram-staining of fresh cells and observation with a transmission microscopy with a color camera using a 50X dry objective (EC Epiplan N.A. 0.75, ZEISS, Oberkochen, Germany), at the Centre for Cellular Imaging (University of Gothenburg). The microscope used was a wide-field, upright optical microscope (Axio Imager.Z2, ZEISS).

Standard biochemical tests (API^®^ ID 32E and API^®^ 20E, bioMérieux) were performed following the manufacturer’s instructions. The results of API^®^ ID 32E were analyzed with the software StrainMatcher^4^ against the CCUG internal database. Additional biochemical tests were performed in conventional tube media (prepared by the Substrate Unit, Department of Clinical Microbiology, Sahlgrenska University Hospital), as described earlier (Bergey et al., 1994; Murray, 2003).

### Cell Fatty Acid-Fatty Acid Methyl Ester Analysis

The strain CCUG 66741^T^ was cultivated on Columbia Blood Agar at 30°C, aerobically, for 30–48 h. The CFA-FAME profile was determined, using an HP 5890 gas chromatograph (Hewlett-Packard, Palo Alto, CA, United States) using a standardized protocol similar to that of the MIDI Sherlock MIS system (Sasser, 2001), described previously (Zamora et al., 2012).

### Antibiotic Susceptibility Testing

Antibiotic sensitivity against ampicillin, cefotaxime, co-trimoxazole, meropenem, tetracycline, tobramycin, and ciprofloxacin, respectively, was tested using disk diffusion method (EUCAST method) using disks from bioMérieux.

### Partial Gene Sequence Determination: PCR and Sanger Sequencing

The total genomic DNA was extracted using a previously described “heat-shock” protocol (Welinder-Olsson et al., 2000) followed by PCR-amplification and determination of 16S rRNA and *dnaJ* partial gene sequences. For 16S rDNA amplification, primers 16F28 and 16R1494 were used (Hauben et al., 1997). Sequencing was performed using the primer 16R806 (Fredricks and Relman, 1998). For *dnaJ* amplification and sequencing, primers DN1-1F and DN1-2R were used (Pham et al., 2007). PCR-products were purified using a PCR/DNA Clean-Up Purification Kit (EURx Ltd., Gdańsk, Poland) and sequenced using a 3730*xl* DNA analyzer system (Applied Biosystems, Foster City, CA, United States). Partial sequences were analyzed with FASTA (Pearson and Lipman, 1988) against the EMBL-EBI Database release Version 36.3.6 (Harrison et al., 2019).

### Whole Genome Sequencing and Genome Assembly

The strains were cultivated on Columbia Blood Agar Base plus 5% defibrinated horse blood, at 30 or 37°C, for 24 h. Genomic DNA was extracted from fresh biomass, using a MagNA Pure Compact Nucleic Acid Isolation Kit I (Roche Diagnostics, Mannheim, Germany) on a MagNA Pure Compact instrument (Roche Diagnostics) following the manufacturer’s instructions. The whole genome sequencing was carried out using Ion Torrent PGM platform (Thermo Fisher Scientific, Waltham, MA, United States), Ion PGM^TM^ Hi-Q^TM^ View Chef 400 Kit and an Ion PGM^TM^ Hi-Q^TM^ View Sequencing Kit (Thermo Fisher Scientific). The raw sequences were trimmed and assembled *de novo* using CLC Genomics Workbench versions 8 and 11 (CLC bio, Aarhus, Denmark). Assembly quality was assessed using QUAST version 3.1 (Gurevich et al., 2013). The genome sequences were deposited in DDBJ/ENA/GenBank and annotated using the NCBI Prokaryotic Genome Annotation Pipeline (PGAP) (Tatusova et al., 2016).

### Search of Additional Genome Sequences

Additional genome sequences of *S. goeteborgense* were searched in GenBank by analyzing house-keeping gene sequences of *S. goeteborgense* CCUG 66741^T^ (16S rRNA, *atpD*, *dnaJ*, *infB*, *gyrB, rpoB*), using BLAST (Altschul et al., 1990), against the “Nucleotide collection (nr/nt)” database. To confirm the species identity, genome sequences of resulting closely-related strains were analyzed by ANIb (Goris et al., 2007), using JSpeciesWS (Richter et al., 2016).

### Full Length 16S rRNA Gene Sequence Similarity

A complete 16S rRNA gene sequence was extracted from the genome sequence of strain CCUG 66741^T^, using Unipro UGENE version 1.17.0 (Okonechnikov et al., 2012). Subsequently, the sequence was analyzed with the “Identify” service of EzBiocloud (Yoon et al., 2017). Type strains showing 16S rRNA gene sequence similarity ≥ 98.7%, the currently accepted species similarity threshold of 16S rRNA gene sequences (Stackebrandt and Ebers, 2006), were selected for calculation of overall genome related indices (i.e., *in silico* DNA-DNA hybridization and ANIb analysis), as recommended by Chun et al. (2018).

### *In silico* DNA-DNA Hybridization

Digital DNA-DNA hybridizations were performed using the webserver Genome-to-Genome Distance Calculator (GGDC) version 2.1 (Meier-Kolthoff et al., 2013), with BLAST + as local alignment tool, as recommended. The results of the recommended formula 2 (“*sum of all identities found in HSPs divided by overall HSP length*”) were considered. This formula is independent of the size of the genome sequences, which makes it robust even if incomplete draft genome sequences are used. Genome-to-Genome Distance was calculated between the genome sequences of strain CCUG 66741^T^ and the genome sequences of the type strains that showed 16S rRNA gene sequence similarity ≥ 98.7% (Supplementary Table S3).

### Average Nucleotide Identity (ANI)

The ANIb values (Goris et al., 2007) between the genome sequence of strain CCUG 66741^T^ and the genome sequences of the type strains were calculated using JSpeciesWS (Richter et al., 2016). With this approach (ANIb), the “query” genome sequence is split into fragments of 1,020 nt, which are then analyzed with the BLASTN algorithm (Altschul et al., 1990) against the “reference” genome sequence. For each pairwise comparison, ANIb analyses are calculated bi-directionally.

### Multilocus Sequence Analysis (MLSA)

The family *Enterobacteriaceae* is currently formed by 32 genera with validly published names. The genome sequences of the type strains of the type species of 30 genera with validly published names of the family were downloaded from GenBank (Supplementary Table S4), including *L. nimipressuralis* CCUG 25894^T^, determined in this study. For MLSA, the complete sequences of six house-keeping genes (16S rRNA, *atpD*, *dnaJ*, *infB*, *gyrB, rpoB*) were extracted from the 30 genome sequences and from the genome sequence of strain CCUG 66741^T^ using Unipro UGENE version 1.17.0 (Okonechnikov et al., 2012). The extracted sequences were imported into CLC Genomics Workbench version 11 (CLC bio), aligned, and a distance matrix with similarity percentage values was created for each gene. If a gene sequence was not found in a genome sequence, GenBank was screened for additional genome sequences of that type strain or partial sequences of the respective gene. Moreover, an MLSA-based phylogenetic tree was constructed, based on concatenated sequence alignments of *atpD*, *infB*, *gyrB*, and *rpoB* gene sequences, including partial sequences of the type strain of *Pluralibacter pyrinus*, the other species with validly published name of the genus *Pluralibacter* (GenBank accession numbers: JX424878, JX425008, JX425137, JX425267). Additionally, nearly-complete 16S rRNA gene sequences were obtained from GenBank for the four type strains of type species that did not have publicly available genome sequence. Once imported, the 16S rRNA gene sequences were aligned and a phylogenetic tree was constructed. In both cases, distances were calculated by the Jukes-Cantor method (Jukes and Cantor, 1969) and the phylogenetic trees were constructed using the neighbor-joining method (Saitou and Nei, 1987) and bootstrap analysis (1,000 replicates) (Felsenstein, 1985). The phylogenetic trees were displayed on-line with the Interactive Tree Of Life (iTOL) (Letunic and Bork, 2016).

### ANI Dendrogram

ANIb values were calculated using JSpeciesWS (Richter et al., 2016) between all the 30 genome sequences of type strains of type species of the family *Enterobacteriaceae* and the genome sequences of strains CCUG 66741^T^, BIGb0156 and YR018, all strains vs. all strains (“all-vs.-all”). Subsequently, the matrix containing all the ANIb values was used to construct a dendrogram with the software PermutMatrix v1.9.4 (Caraux and Pinloche, 2005), using a Pearson’s distance correlation and a hierarchical clustering with an average linkage method (UPGMA). The dendrogram was visualized on-line with the Interactive Tree Of Life (iTOL) (Letunic and Bork, 2016).

### Core Genome-Based Phylogeny

A core genome-based phylogenomic analysis was performed with all the available genome sequences of type strains of type species of genera with validly published names of the family *Enterobacteriaceae* (Supplementary Table 4) and the genome sequences of strains CCUG 66741^T^, BIGb0156, and YR18. The genome sequences were annotated with Prokka v1.11 (Seemann, 2014). All the amino acid sequences were compared by BLASTP (all VS all) with the software GET_HOMOLOGUES v07022017 (Contreras-Moreira and Vinuesa, 2013). The protein sequences were clustered following a 70/70 criterion (i.e., 70% of identity over 70% of the length), using three clustering algorithms: BDBH, COGtriangles (Kristensen et al., 2010) and OrthoMCL (Li et al., 2003). The intersection of the three algorithms was used to determine a consensus core genome. The amino acid sequences of the single copy genes of the consensus core genome were aligned with Clustal Omega v1.2.0 (Sievers et al., 2011). Poorly aligned regions were eliminated with Gblocks v0.91 (Castresana, 2000). The trimmed alignments were concatenated and a phylogenetic tree was constructed with PhyML v3.1 (Guindon et al., 2010), using a Shimodaira-Hasegawa-like approximate likelihood-ratio test (SH-aLRT) for branching statistical support (Anisimova and Gascuel, 2006). The phylogenetic tree was displayed on-line with the Interactive Tree Of Life (iTOL) (Letunic and Bork, 2016).

### Virulence Genes and Characterization of Novel *qnrB* Variant

The genome sequence of *S. goeteborgense* CCUG 66741^T^ was assessed for pathogenic potential by PathogenFinder^5^, a tool developed for predicting the pathogenicity of newly sequenced strains (Cosentino et al., 2013). A novel *qnrB* variant (proposed *qnrB96*) was detected in the genome of strain. A phylogenetic tree of all the *qnrB* variants was constructed using online platform phylogeny.fr (Dereeper et al., 2008), using PhyML v3.1 (Guindon et al., 2010), with bootstrap analysis (1,000 replicates). To test the functionality, the *qnrB96* gene was synthesized at Thermo Fisher Scientific using their GeneArt^®^ Gene Synthesis service and subcloned into the expression vector pZE21-MCS1 as described previously (Marathe et al., 2018). The plasmid *qnrB96*-PZE21 was then transformed into *E. coli* C600Z1 (Expressys, Bammental, Germany) by electroporation. The minimum inhibitory concentrations of ciprofloxacin for the *E. coli* strains expressing *qnrB96* were determined using *E*-tests (bioMérieux) on Mueller-Hinton Agar plates with the addition of 100 ng/μl anhydrotetracycline as an inducer of the expression. *E. coli* strain containing pZE21-MCS1 was used as a negative control. The strain containing plasmid *qnrB1*-PZE21 was used as a positive control.

## Data Availability Statement

The genome sequences of *Scandinavium goeteborgense* CCUG 66741^T^ (= CECT 9823^T^ = NCTC 14286^T^), *Buttiauxella izardii* CCUG 35510^T^, and *Lelliottia nimipressuralis* CCUG 25894^T^ have been deposited in DDBJ/ENA/GenBank under the accession numbers LYLP00000000, QZWH00000000, and SDDX00000000, respectively. The raw sequencing data generated in this study has been deposited in NCBI Sequence Read Archive (SRA) under the accession numbers SRR8561413, SRR8581234, SRR8581235, and SRR8581406. The complete sequences of the 16S rRNA gene and the *qnrB96* gene variant of *S. goeteborgense* CCUG 66741^T^ have been deposited in DDBJ/ENA/GenBank under the accession numbers MK558235 and MK561856, respectively.

## Author Contributions

NM, EM, LS-S, and HJ conceived the study. NM, FS-S, RK, and HJ designed the experiments. NM, FS-S, RK, EM, LS-S, and HJ performed the experiments and analyzed the data. NM, FS-S, EM, LS-S, and HJ drafted the manuscript. DL and RK provided critical inputs. DL and EM acquired the project funding. HJ was responsible for the overall direction of the project. All authors read and approved the final manuscript.

## Conflict of Interest

RK was affiliated with a company, Nanoxis Consulting AB. The company did not have influence on the conception, elaboration and decision to submit the present research article. The remaining authors declare that the research was conducted in the absence of any commercial or financial relationships that could be construed as a potential conflict of interest.

## Footnotes

1 https://www.ncbi.nlm.nih.gov/genbank/

2 www.bacterio.net

3 https://www.dsmz.de/services/online-tools/prokaryotic-nomenclature-up-to-date

4 https://ccug.se/identification/strainmatcher

5 https://cge.cbs.dtu.dk/services/PathogenFinder/

## Supplementary Material

FIGURE S1 MLSA-based phylogenetic tree based on *atpD*, *infB*, *gyrB*, and *rpoB* gene sequences of strains of *S. goeteborgense*, the type strains of the type species of the genera of the family *Enterobacteriaceae*, and the type strain of *P. pyrinus*. The distances were calculated with the Jukes-Cantor method and the tree was constructed using the neighbor-joining method. The numbers at the nodes indicate the bootstrap values (in percentage) from 1,000 replicates. Only bootstrap percentages ≥ 50% are shown.

FIGURE S2 Phylogenetic tree of *qnrB* variants. The numbers at the nodes indicate bootstrap values (scale from 0 to 1) from 1,000 replicates.

TABLE S1 Phenotypic characteristics of strain CCUG 66741^T^ and type strains of type species of related genera of the family *Enterobacteriaceae*.

TABLE S2 Assembly statistics for the whole genome sequence of strain CCUG 66741^T^ and the other type strains of the family *Enterobacteriaceae* sequenced in the present study.

TABLE S3 Sequence similarity values of the complete 16S rRNA gene sequence (1,552 bp) of strain CCUG 66741^T^ with type strains, using the “Identify” engine of EzBioCloud and ANIb and *in silico* DNA-DNA hybridization (DDH) values between the genome sequence of strain CCUG 66741^T^ and the genome sequences of the type strains showing 16S rRNA gene sequence similarity values ≥ 98.7% (shaded in green). Species with 16S rRNA gene sequence similarity < 98.7% are shaded in red.

TABLE S4 List of type species of the genera with validly published names of the family *Enterobacteriaceae*. GenBank accession numbers for the genome sequences and the 16S rRNA gene sequences (if available) of the type strains of the type species are shown.

TABLE S5 Average nucleotide identity (ANIb) and percent identity values for six house-keeping genes (16S rRNA, *atpD*, *dnaJ*, *infB*, *gyrB*, *rpoB*) of the strain CCUG 66741^T^ compared to the type strains of the type species of the genera of the family *Enterobacteriaceae*. Green shadings indicate the three highest values of each column.

## Abbreviations

ANI: average nucleotide identity
ANIb: average nucleotide identity based on BLAST
CFA-FAME: cell fatty acid-fatty acid methyl ester
MALDI-TOF MS: Matrix-Assisted Laser Desorption/Ionization-Time Of Flight Mass Spectrometry
MLSA: multilocus sequence analysis
*qnr*: quinolone resistance
WGS: whole genome sequencing.

## References

[B1] Adeolu M. Alnajar S. Naushad S. Gupta R. S. (2016). Genome-based phylogeny and taxonomy of the ‘*Enterobacteriales*’: proposal for *Enterobacterales* ord. nov. divided into the families *Enterobacteriaceae*, *Erwiniaceae* fam. nov., *Pectobacteriaceae* fam. nov., *Yersiniaceae* fam. nov., *Hafniaceae* fam. nov., *Morganellaceae* fam. nov., and *Budviciaceae* fam. nov. *Int. J. Syst. Evol. Microbiol.* 66 5575–5599. 10.1099/ijsem.0.001485 27620848

[B2] Albornoz E. Tijet N. De Belder D. Gomez S. Martino F. Corso A. (2017). *qnrE1*, a member of a new family of plasmid-located quinolone resistance genes, originated from the chromosome of *Enterobacter* species. *Antimicrobial. Agents Chemother.* 61 e2555–e2516. 10.1128/AAC.02555-16 28193666 PMC5404601

[B3] Alnajar S. Gupta R. S. (2017). Phylogenomics and comparative genomic studies delineate six main clades within the family *Enterobacteriaceae* and support the reclassification of several polyphyletic members of the family. *Infect Genet. Evol.* 54 108–127. 10.1016/j.meegid.2017.06.024 28658607

[B4] Altschul S. F. Gish W. Miller W. Myers E. W. Lipman D. J. (1990). Basic local alignment search tool. *J. Mol. Biol.* 215 403–410. 10.1016/S0022-2836(05)80360-80362 2231712

[B5] Anisimova M. Gascuel O. (2006). Approximate likelihood-ratio test for branches: a fast, accurate, and powerful alternative. *Syst. Biol.* 55 539–552. 10.1080/10635150600755453 16785212

[B6] Bergey D. Holt J. Krieg N. Sneath P. (1994). *Bergey’s Manual of Determinative Bacteriology.* Williams and Wilkins: Baltimore.

[B7] Brady C. Cleenwerck I. Venter S. Coutinho T. De Vos P. (2013). Taxonomic evaluation of the genus *Enterobacter* based on multilocus sequence analysis (MLSA): proposal to reclassify E. *Nimipressuralis* and E. amnigenus into *Lelliottia* gen. nov. as *Lelliottia nimipressuralis* comb. nov. and *Lelliottia amnigena* comb. nov., respectively, E. gergoviae and E. pyrinus into *Pluralibacter* gen. nov. as *Pluralibacter gergoviae* comb. nov. and *Pluralibacter pyrinus* comb. nov., respectively, E. cowanii, E. radicincitans, E. oryzae and E. arachidis into Kosakonia gen. nov. as *Kosakonia cowanii* comb. nov., *Kosakonia radicincitans* comb. nov., *Kosakonia oryzae* comb. nov. and *Kosakonia arachidis* comb. nov., respectively, and E. turicensis, E. helveticus and E. pulveris into Cronobacter as *Cronobacter zurichensis* nom. nov., *Cronobacter helveticus* comb. nov. and *Cronobacter pulveris* comb. nov., respectively, and emended description of the genera *Enterobacter* and *Cronobacter*. *Syst. Appl. Microbiol.* 36 309–319. 10.1016/j.syapm.2013.03.005 23632228

[B8] Brady C. Cleenwerck I. Venter S. Vancanneyt M. Swings J. Coutinho T. (2008). Phylogeny and identification of *Pantoea* species associated with plants, humans and the natural environment based on multilocus sequence analysis (MLSA). *Syst. Appl. Microbiol.* 31 447–460. 10.1016/j.syapm.2008.09.004 19008066

[B9] Carattoli A. (2009). Resistance plasmid families in *Enterobacteriaceae*. *Antimicrob. Agents Chemother.* 53 2227–2238. 10.1128/aac.01707-08 19307361 PMC2687249

[B10] Caraux G. Pinloche S. (2005). PermutMatrix: a graphical environment to arrange gene expression profiles in optimal linear order. *Bioinformatics* 21 1280–1281. 10.1093/bioinformatics/bti141 15546938

[B11] Castresana J. (2000). Selection of conserved blocks from multiple alignments for their use in phylogenetic analysis. *Mol. Biol. Evol.* 17 540–552. 10.1093/oxfordjournals.molbev.a026334 10742046

[B12] Chun J. Oren A. Ventosa A. Christensen H. Arahal D. R. da Costa M. S. (2018). Proposed minimal standards for the use of genome data for the taxonomy of prokaryotes. *Int. J. Syst. Evol. Microbiol.* 68 461–466. 10.1099/ijsem.0.002516 29292687

[B13] Chun J. Rainey F. A. (2014). Integrating genomics into the taxonomy and systematics of the *Bacteria* and *Archaea*. *Int. J. Syst. Evol. Microbiol.* 64(Pt 2), 316–324. 10.1099/ijs.0.054171-54170 24505069

[B14] Contreras-Moreira B. Vinuesa P. (2013). GET_HOMOLOGUES, a versatile software package for scalable and robust microbial pangenome analysis. *Appl. Environ. Microbiol.* 79 7696–7701. 10.1128/AEM.02411-2413 24096415 PMC3837814

[B15] Cosentino S. Voldby Larsen M. Møller Aarestrup F. Lund O. (2013). PathogenFinder - Distinguishing friend from foe using bacterial whole genome sequence data. *PLoS One* 8:e77302. 10.1371/journal.pone.0077302 24204795 PMC3810466

[B16] Dereeper A. Guignon V. Blanc G. Audic S. Buffet S. Chevenet F. (2008). Phylogeny. fr: robust phylogenetic analysis for the non-specialist. *Nucleic Acids Res.* 36(suppl_2), W465–W469. 18424797 10.1093/nar/gkn180 PMC2447785

[B17] Ee R. Madhaiyan M. Ji L. Lim Y. -L. Nor N. M. Tee K. -K. (2016). *Chania multitudinisentens* gen. nov., sp. nov., an N-acyl-homoserine-lactone-producing bacterium in the family *Enterobacteriaceae* isolated from landfill site soil. *Int. J. Syst. Evol. Microbiol.* 66 2297–2304. 10.1099/ijsem.0.001025 26978486

[B18] Felsenstein J. (1985). Confidence limits on phylogenies: an approach using the bootstrap. *Evolution* 39 783–791. 10.1111/j.1558-5646.1985.tb00420.x 28561359

[B19] Fredricks D. N. Relman D. A. (1998). Improved amplification of microbial DNA from blood cultures by removal of the PCR inhibitor sodium polyanetholesulfonate. *J. Clin. Microbiol.* 36 2810–2816. 9738025 10.1128/jcm.36.10.2810-2816.1998 PMC105069

[B20] Goris J. Konstantinidis K. T. Klappenbach J. A. Coenye T. Vandamme P. Tiedje J. M. (2007). DNA-DNA hybridization values and their relationship to whole-genome sequence similarities. *Int. J. Syst. Evol. Microbiol.* 57(Pt 1), 81–91. 10.1099/ijs.0.64483-64480 17220447

[B21] Guindon S. Dufayard J. F. Lefort V. Anisimova M. Hordijk W. Gascuel O. (2010). New algorithms and methods to estimate maximum-likelihood phylogenies: assessing the performance of PhyML 3.0. *Syst. Biol.* 59 307–321. 10.1093/sysbio/syq010 20525638

[B22] Gurevich A. Saveliev V. Vyahhi N. Tesler G. (2013). QUAST: quality assessment tool for genome assemblies. *Bioinformatics* 29 1072–1075. 10.1093/bioinformatics/btt086 23422339 PMC3624806

[B23] Harrison P. W. Alako B. Amid C. Cerdeño-Tárraga A. Cleland I. Holt S. (2019). The european nucleotide archive in 2018. *Nucleic Acids Res.* 47 D84–D88. 10.1093/nar/gky1078 30395270 PMC6323982

[B24] Hata H. Natori T. Mizuno T. Kanazawa I. Eldesouky I. Hayashi M. (2016). Phylogenetics of family *Enterobacteriaceae* and proposal to reclassify *Escherichia hermannii* and *Salmonella subterranea* as *Atlantibacter hermannii* and *Atlantibacter subterranea* gen. nov., comb. nov. *Microbiol. Immunol.* 60 303–311. 10.1111/1348-0421.12374 26970508

[B25] Hauben L. Vauterin L. Swings J. Moore E. R. (1997). Comparison of 16S ribosomal DNA sequences of all *Xanthomonas* species. *Int. J. Syst. Bacteriol.* 47 328–335. 10.1099/00207713-47-2-328 9103617

[B26] Jacoby G. A. Griffin C. M. Hooper D. C. (2011). *Citrobacter* spp. as a source of qnrB alleles. *Antimicrobial Agents Chemother.* 55 4979–4984. 10.1128/AAC.05187-11 21844311 PMC3195048

[B27] Jukes T. H. Cantor C. R. (1969). “CHAPTER 24 - evolution of protein molecules A2, Munro H.N in *Mammalian Protein Metabolism*, (Cambridge: Academic Press), 21–132. 10.1016/b978-1-4832-3211-9.50009-7

[B28] Kämpfer P. McInroy J. A. Glaeser S. P. (2015). *Enterobacter muelleri* sp. nov., isolated from the rhizosphere of Zea mays. *Int. J. Syst. Evol. Microbiol.* 65 4093–4099. 10.1099/ijsem.0.000547 26294947

[B29] Kämpfer P. Ruppel S. Remus R. (2005). *Enterobacter radicincitans* sp. nov., a plant growth promoting species of the family *Enterobacteriaceae*. *Syst. Appl. Microbiol.* 28 213–221. 10.1016/j.syapm.2004.12.007 15900968

[B30] Kristensen D. M. Kannan L. Coleman M. K. Wolf Y. I. Sorokin A. Koonin E. V. (2010). A low-polynomial algorithm for assembling clusters of orthologous groups from intergenomic symmetric best matches. *Bioinformatics* 26 1481–1487. 10.1093/bioinformatics/btq229 20439257 PMC2881409

[B31] Letunic I. Bork P. (2016). Interactive tree of life (iTOL) v3: an online tool for the display and annotation of phylogenetic and other trees. *Nucleic Acids Res.* 44 W242–W245. 10.1093/nar/gkw290 27095192 PMC4987883

[B32] Li C. Y. Zhou Y. L. Ji J. Gu C. T. (2016). Reclassification of *Enterobacter oryziphilus* and *Enterobacter oryzendophyticus* as *Kosakonia oryziphila* comb. nov. and *Kosakonia oryzendophytica* comb. nov. *Int. J. Syst. Evol. Microbiol.* 66 2780–2783. 10.1099/ijsem.0.001054 27045188

[B33] Li L. Stoeckert C. J. Jr Roos D. S. (2003). OrthoMCL: identification of ortholog groups for eukaryotic genomes. *Genome Res.* 13 2178–2189. 10.1101/gr.1224503 12952885 PMC403725

[B34] Madeira F. Lee J. Buso N. Gur T. Madhusoodanan N. Basutkar P. (2019). The EMBL-EBI search and sequence analysis tools APIs in 2019. *Nucleic acids Res.* 47 W636–W641 30976793 10.1093/nar/gkz268 PMC6602479

[B35] Mahato N. K. Gupta V. Singh P. Kumari R. Verma H. Tripathi C. (2017). Microbial taxonomy in the era of OMICS: application of DNA sequences, computational tools and techniques. *Antonie Van Leeuwenhoek* 110 1357–1371. 10.1007/s10482-017-0928-921 28831610

[B36] Marathe N. P. Janzon A. Kotsakis S. D. Flach C. -F. Razavi M. Berglund F. (2018). Functional metagenomics reveals a novel carbapenem-hydrolyzing mobile beta-lactamase from Indian river sediments contaminated with antibiotic production waste. *Environ. Int.* 112 279–286. 10.1016/j.envint.2017.12.036 29316517

[B37] Meier-Kolthoff J. P. Auch A. F. Klenk H. P. Goker M. (2013). Genome sequence-based species delimitation with confidence intervals and improved distance functions. *BMC Bioinformatics* 14:60. 10.1186/1471-2105-14-60 23432962 PMC3665452

[B38] Mlaga K. Lotte R. Montaudié H. Rolain J. -M. Ruimy R. (2017). ‘*Nissabacter archeti*’ gen. nov., sp. nov., a new member of *Enterobacteriaceae* family, isolated from human sample at Archet 2 hospital, nice, france. *New Microb. New Infect.* 17 81–83. 10.1016/j.nmni.2017.02.001 28392923 PMC5377011

[B39] Murray P. (2003). *Specimen Collection, Transport & Processing: Bacteriology. Manual of Clinical Microbiology.* Washington, DC: ASM Press.

[B40] Naum M. Brown E. W. Mason-Gamer R. J. (2008). Is 16S rDNA a reliable phylogenetic marker to characterize relationships below the family level in the *Enterobacteriaceae*? *J. Mol. Evol.* 66 630–642. 10.1007/s00239-008-9115-3 18504519

[B41] Okonechnikov K. Golosova O. Fursov M. Team U. (2012). Unipro UGENE: a unified bioinformatics toolkit. *Bioinformatics* 28 1166–1167. 10.1093/bioinformatics/bts091 22368248

[B42] Pearson W. R. Lipman D. J. (1988). Improved tools for biological sequence comparison. *Proc. Natl. Acad. Sci. U.S.A.* 85 2444–2448. 10.1073/pnas.85.8.2444 3162770 PMC280013

[B43] Pham H. N. Ohkusu K. Mishima N. Noda M. Monir Shah M. Sun X. (2007). Phylogeny and species identification of the family *Enterobacteriaceae* based on *dnaJ* sequences. *Diagn Microbiol. Infect. Dis.* 58 153–161. 10.1016/j.diagmicrobio.2006.12.019 17368802

[B44] Potter R. F. D’Souza A. W. Wallace M. A. Shupe A. Patel S. Gul D. (2018). Superficieibacter electus gen. nov., sp. nov., an extended-spectrum β-lactamase possessing member of the enterobacteriaceae family, isolated from intensive care unit surfaces. *Front. Microbiol.* 9:1629 10.3389/fmicb.2018.01629 PMC6062592 30079059

[B45] Quainoo S. Coolen J. P. van Hijum S. A. Huynen M. A. Melchers W. J. van Schaik W. (2017). Whole-genome sequencing of bacterial pathogens: the future of nosocomial outbreak analysis. *Clin. Microbiol. Rev.* 30 1015–1063. 10.1128/CMR.00016-17 28855266 PMC5608882

[B46] Ramasamy D. Mishra A. K. Lagier J. C. Padhmanabhan R. Rossi M. Sentausa E. (2014). A polyphasic strategy incorporating genomic data for the taxonomic description of novel bacterial species. *Int. J. Syst. Evol. Microbiol.* 64(Pt 2), 384–391. 10.1099/ijs.0.057091-57090 24505076

[B47] Ribeiro T. G. Novais Â. Branquinho R. Machado E. Peixe L. (2015). Phylogeny and comparative genomics unveil independent diversification trajectories of *qnrB* and genetic platforms within particular *Citrobacter* species. *Antimicrobial Agents Chemother.* 59 5951–5958. 10.1128/aac.00027-15 26169406 PMC4576110

[B48] Richter M. Rosselló-Móra R. (2009). Shifting the genomic gold standard for the prokaryotic species definition. *Proc. Natl. Acad. Sci. U.S.A.* 106 19126–19131. 10.1073/pnas.0906412106 19855009 PMC2776425

[B49] Richter M. Rosselló-Móra R. Oliver Glöckner F. Peplies J. (2016). JSpeciesWS: a web server for prokaryotic species circumscription based on pairwise genome comparison. *Bioinformatics* 32 929–931. 10.1093/bioinformatics/btv681 26576653 PMC5939971

[B50] Saitou N. Nei M. (1987). The neighbor-joining method: a new method for reconstructing phylogenetic trees. *Mol. Biol. Evol.* 4 406–425. 10.1093/oxfordjournals.molbev.a040454 3447015

[B51] Samuel B. S. Rowedder H. Braendle C. Félix M. A. Ruvkun G. (2016). *Caenorhabditis elegans* responses to bacteria from its natural habitats. *Proc. Natl. Acad. Sci. U.S.A.* 113 E3941–E3949. 10.1073/pnas.1607183113 27317746 PMC4941482

[B52] Sasser M. (2001). *Identification of Bacteria by Gas Chromatography of Cellular Fatty Acids.* Newark, DE: MIDI Inc.

[B53] Schürch A. Arredondo-Alonso S. Willems R. Goering R. V. (2018). Whole genome sequencing options for bacterial strain typing and epidemiologic analysis based on single nucleotide polymorphism versus gene-by-gene–based approaches. *Clin. Microbiol. Infect.* 24 350–354. 10.1016/j.cmi.2017.12.016 29309930

[B54] Seemann T. (2014). Prokka: rapid prokaryotic genome annotation. *Bioinformatics* 30 2068–2069. 10.1093/bioinformatics/btu153 24642063

[B55] Sievers F. Wilm A. Dineen D. Gibson T. J. Karplus K. Li W. (2011). Fast, scalable generation of high-quality protein multiple sequence alignments using clustal omega. *Mol. Syst. Biol.* 7:539. 10.1038/msb.2011.75 21988835 PMC3261699

[B56] Stackebrandt E. Ebers J. (2006). Taxonomic parameters revisited: tarnished gold standards. *Microbiol. Today* 33 152–155.

[B57] Stephan R. Grim C. J. Gopinath G. R. Mammel M. K. Sathyamoorthy V. Trach L. H. (2014). Re-examination of the taxonomic status of *Enterobacter helveticus*, *Enterobacter pulveris* and *Enterobacter turicensis* as members of the genus *Cronobacter* and their reclassification in the genera *Franconibacter* gen. nov. and *Siccibacter* gen. nov. as *Franconibacter helveticus* comb. nov., *Franconibacter pulveris* comb. nov. and *Siccibacter turicensis* comb. nov., respectively. *Int. J. Syst. Evol. Microbiol.* 64(Pt 10):3402. 10.1099/ijs.0.059832-0 25028159 PMC4179279

[B58] Strahilevitz J. Jacoby G. A. Hooper D. C. Robicsek A. (2009). Plasmid-mediated quinolone resistance: a multifaceted threat. *Clin. Microbiol. Rev.* 22 16–19. 10.1128/cmr.00016-19 PMC2772364 19822894

[B59] Tatusova T. DiCuccio M. Badretdin A. Chetvernin V. Nawrocki E. P. Zaslavsky L. (2016). NCBI prokaryotic genome annotation pipeline. *Nucleic acids Res.* 44 6614–6624. 10.1093/nar/gkw569 27342282 PMC5001611

[B60] Welinder-Olsson C. Kjellin E. Badenfors M. Kaijser B. (2000). Improved microbiological techniques using the polymerase chain reaction and pulsed-field gel electrophoresis for diagnosis and follow-up of enterohaemorrhagic *Escherichia coli* infection. *Eur. J. Clin. Microbiol. Infect. Dis.* 19 843–851. 10.1007/s100960000380 11152309

[B61] World Health Organization [WHO] (2017). *List of Bacteria for Which New Antibiotics are Urgently Needed.* Geneva: World Health Organization.

[B62] Yarza P. Spröer C. Swiderski J. Mrotzek N. Spring S. Tindall B.J. (2013). Sequencing orphan species initiative (SOS): filling the gaps in the 16S rRNA gene sequence database for all species with validly published names. *Syst. Appl. Microbiol.* 36 69–73. 10.1016/j.syapm.2012.12.006 23410935

[B63] Yoon S. -H. Ha S. -M. Kwon S. Lim J. Kim Y. Seo H. (2017). Introducing EzBioCloud: a taxonomically united database of 16S rRNA gene sequences and whole-genome assemblies. *Int. J. Syst. Evol. Microbiol.* 67:1613. 10.1099/ijsem.0.001755 28005526 PMC5563544

[B64] Young J. M. Park D. C. (2007). Relationships of plant pathogenic *enterobacteria* based on partial *atpD*, *carA*, and *recA* as individual and concatenated nucleotide and peptide sequences. *Syst. Appl. Microbiol.* 30 343–354. 10.1016/j.syapm.2007.03.002 17451899

[B65] Zamora L. Fernández-Garayzábal J. F. Svensson-Stadler L. A. Palacios M. A. Domínguez L. Moore E. R. (2012). *Flavobacterium oncorhynchi* sp. nov., a new species isolated from rainbow trout (*Oncorhynchus mykiss*). *Syst. Appl. Microbiol.* 35 86–91. 10.1016/j.syapm.2011.11.007 22227311

