# Supplementary figures and images for: Scandinavium goeteborgense gen. nov., sp. nov., a New Member of the Family Enterobacteriaceae Isolated From a Wound Infection, Carries a Novel Quinolone Resistance Gene Variant
Source: Front Microbiol. 2019 Nov 5;10:2511. doi: 10.3389/fmicb.2019.02511 (PMC6856666; doi:10.3389/fmicb.2019.02511)

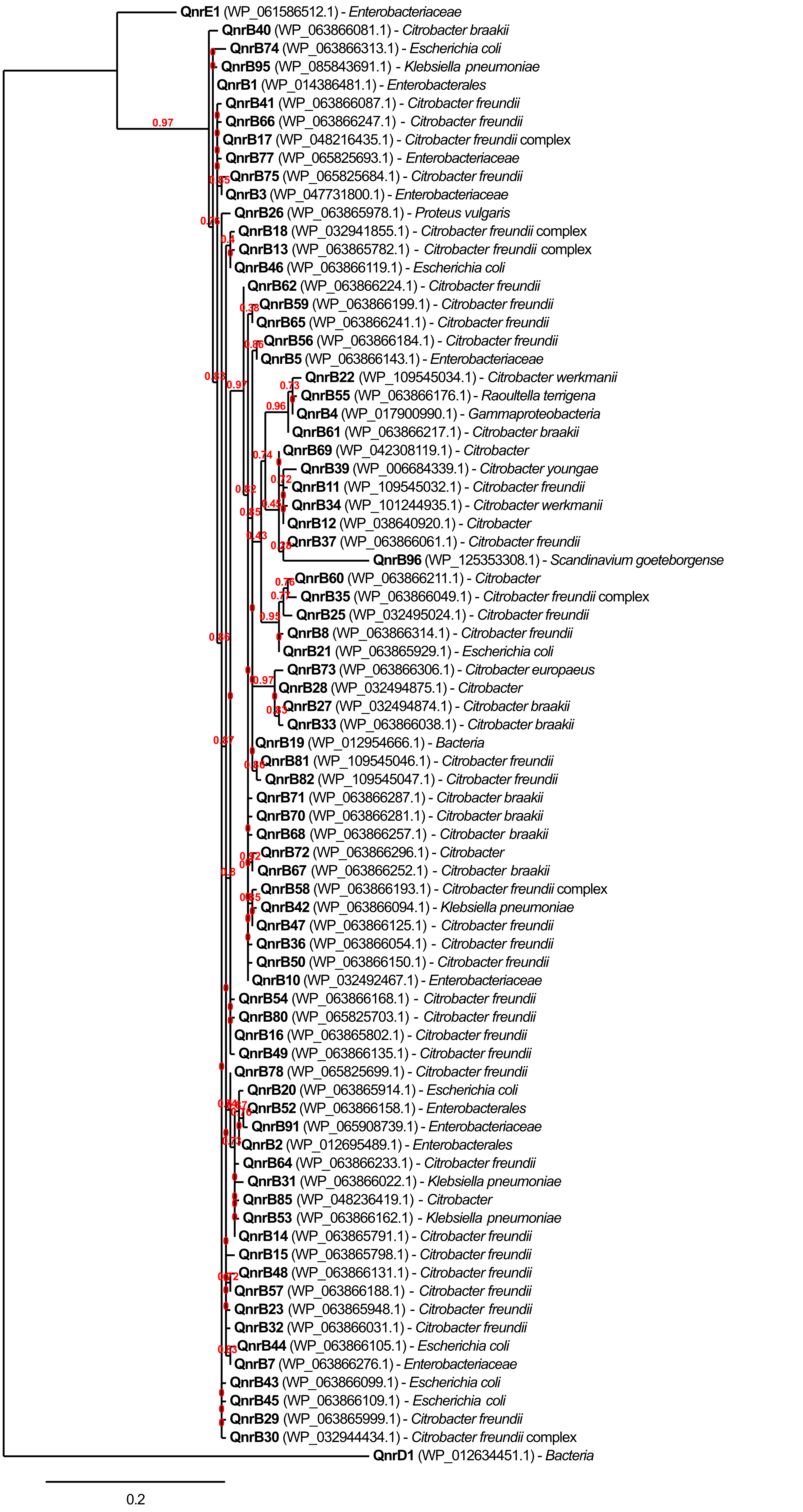

Supplement: FIGURE S2 — Phylogenetic tree of qnrB variants. The numbers at the nodes indicate bootstrap values (scale from 0 to 1) from 1,000 replicates. [file Image_2.tiff]
