# Supplementary material for: Scandinavium goeteborgense gen. nov., sp. nov., a New Member of the Family Enterobacteriaceae Isolated From a Wound Infection, Carries a Novel Quinolone Resistance Gene Variant
Source: Front Microbiol. 2019 Nov 5;10:2511. doi: 10.3389/fmicb.2019.02511 (PMC6856666; doi:10.3389/fmicb.2019.02511)
Supplement: TABLE S2 — Assembly statistics for the whole genome sequence of strain CCUG 66741T and the other type strains of the family Enterobacteriaceae sequenced in the present study. [file Table_2.pdf]

Table S2. Assembly statistics for the whole genome sequence of strain CCUG 66741[T] and the other type strains of the family *Enterobacteriaceae* sequenced in the present study.

Strain	Accession number	Ion Torrent platform	Coverage	No. of contigs	G+C (%)	N_{50} (bp)	Total length (bp)	No. of CDS[a]
Scandinavium goeteborgense CCUG 66741[T]	LYLP00000000	PGM	43x	192	54.31	74,823	4,539,908	4,384
Lelliottia nimipressuralis CCUG 25894[T]	SDDX00000000	S5	36x	67	54.85	236,780	4,616,251	4,446
Buttiauxella izardii CCUG 35510[T]	QZWH00000000	S5	280x	91	51.26	91,315	4,769,981	4,389

a CDS, coding sequences.
