# Supplementary material for: Scandinavium goeteborgense gen. nov., sp. nov., a New Member of the Family Enterobacteriaceae Isolated From a Wound Infection, Carries a Novel Quinolone Resistance Gene Variant
Source: Front Microbiol. 2019 Nov 5;10:2511. doi: 10.3389/fmicb.2019.02511 (PMC6856666; doi:10.3389/fmicb.2019.02511)
Supplement: TABLE S3 — Sequence similarity values of the complete 16S rRNA gene sequence (1,552 bp) of strain CCUG 66741T with type strains, using the “Identify” engine of EzBioCloud and ANIb and in silico DNA-DNA hybridization (DDH) values between the genome sequence of strain CCUG 66741T and the genome sequences of the type strains showing 16S rRNA gene sequence similarity values ≥ 98.7% (shaded in green). Species with 16S rRNA gene sequence similarity < 98.7% are shaded in red. [file Table_3.pdf]

Table S3. Sequence similarity values of the complete 16S rRNA gene sequence (1,552 bp) of strain CCUG 66741^T with type strains, using the 'Identify' engine of EzBioCloud and ANIb and *in silico* DNA-DNA hybridization (DDH) values between the genome sequence of strain CCUG 66741^T and the genome sequences of the type strains showing 16S rRNA gene sequence similarity values ≥ 98.7% (shaded in green). Species with 16S rRNA gene sequence similarity < 98.7% are shaded in red.

Type strain	16S rRNA gene sequence similarity (%)	*In silico* DDH (%)	ANIb (%)	GenBank accession number
Leclercia adecarboxylata NBRC 102595^T	98.8	22.2	78.31	JMPM00000000
Enterobacter ludwigii EN-119^T	98.8	22.0	78.20	CP017279
Enterobacter roggenkampii EN-117^T	98.8	22.5	78.66	CP017184
Pantoea rodasii LMG 26273^T	98.8	19.8	72.57	PIQI00000000
Buttiauxella izardii DSM 9397^T	98.7	20.5	75.61	QZWH00000000
Citrobacter freundii DSM 30039^T	98.6	n/a	n/a	n/a
Buttiauxella agrestis ATCC 33320^T	98.6	n/a	n/a	n/a
Enterobacter cancerogenus ATCC 33241^T	98.6	n/a	n/a	n/a
Buttiauxella noackiae ATCC 51607^T	98.6	n/a	n/a	n/a
Buttiauxella ferragutiae ATCC 51602^T	98.6	n/a	n/a	n/a
Buttiauxella gaviniae ATCC 51604^T	98.6	n/a	n/a	n/a
Klebsiella aerogenes KCTC 2190^T	98.6	n/a	n/a	n/a
Enterobacter asburiae JCM 6051^T	98.6	n/a	n/a	n/a
Lelliottia amnigena NBRC 105700^T	98.6	n/a	n/a	n/a
Enterobacter chuandaensis 090028	98.6	n/a	n/a	n/a
Enterobacter huaxiensis 090008^T	98.5	n/a	n/a	n/a
Kluyvera cryocrescens ATCC 33435^T	98.5	n/a	n/a	n/a
Erwinia aphidicola DSM 19347^T	98.5	n/a	n/a	n/a
Citrobacter werkmanii NBRC 105721^T	98.5	n/a	n/a	n/a
Kluyvera intermedia NBRC 102594^T	98.5	n/a	n/a	n/a
Enterobacter bugandensis EB-247^T	98.5	n/a	n/a	n/a
Lelliottia aquatilis 6331-17^T	98.5	n/a	n/a	n/a
Enterobacter sichuanensis WCHECl1597^T	98.5	n/a	n/a	n/a

Lelliottia jeotgali PFL01^T	98.4	n/a	n/a	n/a
Citrobacter braakii ATCC 51113^T	98.4	n/a	n/a	n/a
Citrobacter portucalensis A60^T	98.4	n/a	n/a	n/a
Citrobacter murliniae CDC 2970-59^T	98.3	n/a	n/a	n/a
Kluyvera ascorbata ATCC 33433^T	98.3	n/a	n/a	n/a
Buttiauxella warmboldiae DSM 9404^T	98.3	n/a	n/a	n/a
Lelliottia nimipressuralis LMG 10245^T	98.3	n/a	n/a	n/a
Buttiauxella brennerae DSM 9396^T	98.2	n/a	n/a	n/a
Pseudescherichia vulneris NBRC 102420^T	98.2	n/a	n/a	n/a
Citrobacter pasteurii CIP 55.13^T	98.2	n/a	n/a	n/a
Yokenella regensburgei ATCC 49455^T	98.2	n/a	n/a	n/a
