# Supplementary material for: Scandinavium goeteborgense gen. nov., sp. nov., a New Member of the Family Enterobacteriaceae Isolated From a Wound Infection, Carries a Novel Quinolone Resistance Gene Variant
Source: Front Microbiol. 2019 Nov 5;10:2511. doi: 10.3389/fmicb.2019.02511 (PMC6856666; doi:10.3389/fmicb.2019.02511)
Supplement: TABLE S4 — List of type species of the genera with validly published names of the family Enterobacteriaceae. GenBank accession numbers for the genome sequences and the 16S rRNA gene sequences (if available) of the type strains of the type species are shown. [file Table_4.pdf]

Table S4. List of type species of the current genera of the family *Enterobacteriaceae* with validly published names. GenBank accession numbers for the genome sequences and the 16S rRNA gene sequences (if available) of the type strains of the type species are shown.

Type species	Type strain genome sequence accession number	Type strain 16S rRNA gene sequence accession number
Biostraticola tofi	SMCR00000000	AM774412
Buttiauxella agrestis	JMPI00000000	JMPI00000000
Cedecea davisae	ATDT00000000	ATDT00000000
Citrobacter freundii	BBMV00000000	BBMV00000000
Cronobacter sakazakii	CP011047	CP011047
Enterobacillus tribolii	QRAP00000000	QRAP00000000
Enterobacter cloacae	CP001918	CP001918
Escherichia coli	AGSE00000000	AGSE00000000
Franconibacter helveticus	AWFX00000000	AXDK00000000
Gibbsiella quercinecans	CP014136	CP014136
Izhakiella capsodis	FOVC00000000	KF436763
Klebsiella pneumoniae	JOOW00000000	JOOW00000000
Kluyvera ascorbata	JMPL00000000	JMPL00000000
Kosakonia cowanii	BBEU00000000	BBEU00000000
Leclercia adecarboxylata	JMPM00000000	BCNP00000000
Lelliottia nimipressuralis	SDDX00000000	SDDX00000000
Mangrovibacter plantisponsor	QGTS00000000	QGTS00000000
Metakosakonia massiliensis	CAEO00000000	CAEO00000000
Phytobacter diazotrophicus	SMDE00000000	KY288669
Plesiomonas shigelloides	LT575468	LT575468
Pluralibacter gergoviae	BCZS00000000	UGTG00000000
Pseudescherichia vulneris	BBMZ00000000	BBMZ00000000
Pseudocitrobacter faecalis	QNRL00000000	QNRL00000000
Raoultella planticola	JMPP00000000	JMPP00000000
Rosenbergiella nectarea	FOGC00000000	FOGC00000000
Saccharobacter fermentatus[a]	---	---
Salmonella enterica	CP019186	CP019186
Shigella dysenteriae	CP026774	CP026774
Shimwellia pseudoproteus	---	FJ267523
Siccibacter turicensis	AWFZ00000000	AVPP00000000
Trabulsiella guamensis	JMTB00000000	JMTB00000000
Yokenella regensburgei	JMPS00000000	JMPS00000000

[a] Type strain not available at any recognized culture collection.
